# Huangqi decoction ameliorates kidney injury in db/db mice by regulating the BMP/Smad signaling pathway

**DOI:** 10.1186/s12906-023-04029-1

**Published:** 2023-06-26

**Authors:** Ying Chen, Rong Rui, Li Wang, Hao Wang, Bingbing Zhu, Aili Cao

**Affiliations:** 1 Department of Nephrology, Laboratory of Renal Disease, Putuo Hospital, Shanghai University of Traditional Chinese Medicine, 164 LanXi Road, Shanghai, 200062 China; 2 Department of Nephrology, Shanghai Sixth People’s Hospital Affiliated to Shanghai Jiao Tong University School of Medicine, Shanghai, 200233 China

**Keywords:** Huangqi decoction (HQD), Diabetic kidney disease (DKD), Lipid metabolism, Db/db mice

## Abstract

**Purpose:**

This study aims to investigate the mechanism underlying the beneficial effects of Huangqi decoction (HQD) on Diabetic kidney disease (DKD) in diabetic db/db mice.

**Methods:**

Eight-week-old male diabetic db/db mice were randomly divided into four groups: Model (1% CMC), HQD-L (0.12 g/kg), HQD-M (0.36 g/kg), and HQD-H (1.08 g/kg) groups. Non-diabetic db/m mice were served as the control group. These mice received HQD treatment for 8 weeks. After treatment, the kidney function, histopathology, micro-assay, and protein expression levels were assessed.

**Results:**

HQD treatment improved the albumin/creatine ratio (ACR) and 24 h urinary albumin excretion, prevented the pathological phenotypes of increased glomerular volume, widened mesangial areas, the of mesangial matrix proliferation, foot process effacement, decreased nephrin expression and reduced number of podocytes. Expression profiling analysis revealed global transcriptional changes that predicted related functions, diseases and pathways. HQD treatment activated protein expressions of BMP2, BMP7, BMPR2, and active-Rap1, while inhibiting Smad1 and phospho-ERK. In addition, HQD was associated with improvements in lipid deposition in the kidneys of db/db mice.

**Conclusion:**

HQD ameliorated the progression of DKD in db/db mice by regulating BMP transcription and downstream targets, inhibiting the phosphorylation of ERK and the expression of Smad1, promoting Rap1 binding to GTP, and regulating the lipid metabolism. These findings provide a potential therapeutic approach for treating DKD.

**Supplementary Information:**

The online version contains supplementary material available at 10.1186/s12906-023-04029-1.

## Background

Diabetes mellitus is a common disease that affects more than 463 million adults globally [1]. Diabetic kidney disease (DKD) is one of the most common and serious chronic microvascular complications of diabetes mellitus and the main cause of end-stage renal disease (ESRD) and death. Early diagnosis of DKD is critical, as the onset of proteinuria increases the risk of progression to ESRD by approximately 14 times [2]. Although angiotensin-converting enzyme inhibitors (ACEIs) and angiotensin receptor blockers (ARBs) are commonly used in the clinical treatment of DKD, they can have adverse effects such as heart failure [3, 4]. There is a pressing need for systematic approaches to improve the treatment of DKD. In addition to conventional therapeutic measures and basic recommendations, such as lifestyle changes, phytotherapy, an alternative branch of medicine, has emerged as a promising tool to support the management of diabetes mellitus and related disorders [5]. Evidence-based studies have shown that active compounds found in plant-based raw materials possess antioxidant, anti-inflammatory, and immunomodulatory properties, making them potentially beneficial in the treatment of DKD [4, 6].

Bone morphogenetic proteins (BMPs) is a member of the transforming growth factor (TGF-β) superfamily, known for its highly conserved functional protein. It was first discovered by Urist from a decalcified bone matrix extract and named BMP due to its ability to induce ectopic bone formation [7, 8]. Current studies have shown that BMP has a wide range of biological functions [9] and plays an instrumental role in kidney development [10]. There are two types of BMP-induced signaling pathways, including the canonical Smad-dependent pathway and Smad-independent pathways [11]. Disruption of the classic Smad-dependent BMP pathway has been linked to accelerated onset and progression of renal fibrosis[12], highlighting the significance of BMP in kidney health.

The Ras-like small GTPase Rap1 functions as a molecular switch by binding GTP or GDP to switch between active and inactivated states. The integrity of the slit diaphragm is directly linked to Rap1 signaling [13]. Previous research has revealed that abnormalities in Rap1GTP in podocytes result in variations in downstream mitogen-activated protein kinase (MAPK) signaling [14]. Furthermore, a recent study has discovered a novel mechanism in pulmonary artery smooth muscle cells, where extracellular regulated protein kinase (ERK), a component of MAPK, can regulate the phosphorylation of Smad, thereby facilitating the entry of BMP into the nucleus [15]. Notably, the activity of Rap1GTP is inversely correlated with the phosphorylation level of ERK [14], suggesting that ERK-mediated downregulation of Rap1GTP activity may play a role in this process.

In most cases, disruptions in lipid metabolism are commonly observed in patients with DKD, and emerging research suggests that BMPs are involved in maintaining metabolic processes [16–19]. Traditional Chinese medicine (TCM), which has been widely used in China for thousands of years. One such TCM formula, Huangqi decoction (HQD), composed of Astragali Radix, Poria, Trichosanthis Radix, Ophiopogonis Radix, Schisandrae Chinensis Fructus, Glycyrrhizae Radix et Rhizoma Praeparata Cum Melle, and Rehmanniae Radix, has shown promising results in regulating DKD through multiple targets and pathways. HQD has been found to inhibit podocyte injury by modulating oxidative stress, endoplasmic reticulum stress, as well as increasing insulin sensitivity, ultimately alleviating DKD [20–25]. These studies found that Astragaloside, one of the main components of HQD, ameliorates DKD and inhibits interstitial renal fibrosis by alleviating endoplasmic reticulum stress [21]. The present investigation aims to further elucidate the mechanism by which HQD protects against renal glomerular dysfunction in DKD, providing novel insights for the treatment of DKD.

## Methods and materials

### HQD preparation

HQD, consisting of Astragali Radix, Poria, Trichosanthis Radix, Ophiopogonis Radix, Schisandrae Chinensis Fructus, Glycyrrhizae Radix et Rhizoma Praeparata Cum Melle, and Rehmanniae Radix (Table 1), were purchased from Shanghai Huayu Chinese Herbs Co. Ltd. (Shanghai, China). All the herbs were powdered and mixed thoroughly to create a homogenous mixture. The medicinal herb mixture was then subjected to triple extraction using four times volumes of water. The resulting extract was concentrated under vacuum to obtain a thick decoction. Ethanol was added to the concentrated decoction to achieve a final ethanol concentration of 70%. After overnight precipitation, the supernatant was collected and dried in an oven at 105 °C for 48 h, resulting in a production rate of 13.3%. HQD was formulated as suspensions in 1% sodium carboxymethylcellulose in ddH_2_O at concentrations of 10, 40, and 110 mg/ml for use in animal studies. Intragastric administration was carried out at doses of 0.12, 0.36, and 1.08 g/kg, respectively.

**Table 1:** Huangqi Decoction composition

Chinese name	English name	Botanical name	Family	Weight (g)	Part used
Huangqi	Astragali Radix	*Astragalus membranaceus* (Fisch.) Bge. var. *mongholicus* (Bge.) Hsiao	Leguminosae	30	Root
Fuling	Poria	*Poria cocos* (Schw.) Wolf	Polyporaceae	30	Sclerotia
Tianhuafen	Trichosanthis Radix	*Trichosanthes kirilowii* Maxim	Cucurbitaceae	30	Root
Maidong	Ophiopogonis Radix	*Ophiopogon japonicus* (L.f) Ker-Gawl	Liliaceae	30	Tuber
Wuweizi	Schisandrae Chinensis Fructus	*Schisandra chinensis* (Turcz.) Baill	Magnoliaceae	15	Fruit
Zhigancao	Glycyrrhizae Radix et Rhizoma Praeparata Cum Melle	*Glycyrrhiza uralensis* Fisch	Leguminosae	15	Root and Rhizome
Dihuang	Rehmanniae Radix	*Rehmannia glutinosa* Libosch	Scrophulariaceae	45	Root

### Identification of the main compounds in HQD

The HQD extract was subjected to comprehensive analysis using LC–MS, following established protocols [26]. The composition of the complete HQD was thoroughly investigated, and the results are presented in the supplementary Figure S2, along with detailed MS/MS analysis elucidating the molecular characteristics of the components identified.

### Animal treatment

All animal protocols in this study were approved by the Ethics Committee of Shanghai University of Traditional Chinese Medicine, and the animal ethics number is PZSHUTCM201225002. Db/db (C57BLKS/J-LepRdb/LepRdb) mice, also known as leptin receptor-deficient mice, are commonly used as a model to study DKD. Db/db mice spontaneously develop obesity and type 2 diabetes due to a mutation in the leptin receptor gene, which leads to insulin resistance and hyperglycemia. Db/db mice exhibit characteristics of DKD, including glomerular hypertrophy, increased urinary albumin excretion, and progressive renal fibrosis, which are known to be involved in the development and progression of DKD in humans [27–29]. In research studies involving db/db mice, db/m (C57BLKS/J-LepRdb/LepRdb/ +) mice are often used as the control group. Db/m mice are typically littermates or genetically similar mice that do not carry the mutation in the leptin receptor gene, and therefore do not develop diabetes or other metabolic disorders like db/db mice. In this study, diabetic db/db mice and nondiabetic db/m control mice at the age of 6 weeks were purchased from the Nanjing Institute of Biomedicine affiliated with Nanjing University (Nanjing, China). Animals were housed in a specific-pathogen-free room in a 12:12 h light–dark cycle and were given free access to water and chow. All the operations and experimental procedures complied with the ethical standard in the Laboratory Animal Guideline for review of animal welfare, The National Standard of the People’s Republic of China (GB/T 35892–2018), the Guide for the Care and Use of Laboratory Animals: Eighth Edition. After two weeks of adaptive housing and feeding, mice were randomly allocated into different groups for specific treatment. Random blood glucose levels were monitored every four weeks by blood samples obtained from the tail vein with a glucometer (Omron HEA-230). Db/db mice were subjected to diabetes through genetic modification. Any animal that died or was severely injured during the experiment was excluded from the study. A total of diabetic db/db mice were divided into 4 groups (n = 5–6) and were administrated either a vehicle (1% sodium carboxymethylcellulose, CMC) as a control, or three different doses of HQD (high dose 1.08 g/kg, medium dose: 0.36 g/kg, low dose: 0.12 g/kg). Additionally, a group of nondiabetic db/m mice received the vehicle. HQD or vehicle was administered to the animals daily in the morning via gavage for 8 consecutive weeks. Urine samples were collected from all mice at the ages of 8, 12, and 16 weeks to measure urinary protein levels. On the last day of week 16, the animals were anesthetized with an intraperitoneal injection of sodium pentobarbital (100 mg/kg) in the morning after overnight fasting and sacrificed to harvest tissues. The left kidney was divided into three parts, one part was subsequently placed into 4% paraformaldehyde for immunohistochemical stains, another part was stored in 2.5% glutaraldehyde for electron microscopy observation and the remaining part was stored in 18% sucrose at 4 °C for immunofluorescence staining. The cortex of the right kidney was stored at -80 °C for protein expression detection.

### Urine albumin and creatinine measurement

Twenty-four-hour urine collections were obtained from mice using metabolic cages to ensure time collection. Urine albumin was measured using an ELISA kit (Bethyl Laboratory, Houston, TX), while urine creatinine was quantified using a colorimetric assay kit (Cayman Chemical, Ann Arbor, MI, USA). albumin-creatinine ratio was expressed as ACR, a widely accepted indicator of kidney function. The reproducibility of this assay was assessed, and the coefficients of variance (CV) were found to be consistently less than 3% when the same sample was measured three time consecutively, indicating high precision and accuracy in the measurements.

### RNA isolation and micro-array analysis

Total RNA was extracted from approximately 50 mg of flash-frozen kidney cortex samples obtained from three treated and three non-treated db/db mice using the Qiazol extraction method, folloed by on-coloumn DNase I treatment (Qiagen, CA, USA). The quality of the extracted RNA was evaluated usinng BioAnalyzer (Agilent, CA, USA), and only samples with RNA integrity number (RIN) scores of 7 or higher were included in the subsequent analysis. Samples was sent to H-Wayen Biotechnologies (Shanghai, China) to detect the expression level of the whole genome mRNA.

### Western blot analysis

The renal cortex was homogenized in RIPA lysis buffer containing 1X protease inhibitors and phosphatase inhibitors (Beyotime, Shanghai, China), and lysates were collected after centrifugation for 10 min at 12,000 g. The concentration of total protein in the tissue lysates was determined using a BCA kit (Pierce, Rockford, IL, USA). Subsequently, 60 µg of lysate protein from each animal was separated by SDS‒PAGE (8%-12%) and transferred to PVDF membranes (Millipore, IPVH00010). Then, the membranes were immunoblotted with primary antibodies against BMP2 (Abcam, Ab214821, 1:1000 dilution), BMP7 (Proteintech, 12,221–1-AP, 1:1000 dilution), BMPR2 (Proteintech, 14,376–1-AP, 1:1000 dilution), phospho-ERK (CST, 4370S, 1:2000 dilution), ERK (CST, 4695S, 1:1000 dilution), P-SMAD1,5,9 (CST, 13,820, 1:1000 dilution), SMAD4 (Affinity, AF5247, 1:500 dilution), SMAD1 (CST, 6944, 1:1000 dilution), CXCL16 (Affinity, DF13312, 1:500 dilution) and ß-actin (Abcam, ab6276, 1:10,000 dilution) after blocking in 5% bovine serum albumin in TBST for 1 h. All primary antibodies were incubated at 4 °C overnight. Then membranes were washed in TBST and incubated with HRP-conjugated secondary antibody from Jackson ImmunoResearch (111–035-003 or 115–035-003) for 1 h at room temperature. To remove the residual secondary antibody, the membranes were washed in TBST and incubated with enhanced chemiluminescence (ECL) from Millipore (WBULS0500). An Image Quant LAS 500 was employed to visualize and capture the blot bands, which were quantified by densitometry using ImageJ.

### Active Rap1 pull down

The assessment of active Rap1 levels in kidney cortex was conducted using a pull-down assay, which involved the use of GST-tagged fusion proteins. These fusion proteins were designed to encompass amino acids 788–884 of the human RalGDS-RAP-binding structural domain, and were immobilized on glutathione agarose beads. The pull-down assay was performed utilizing the Active Rap1 Pull-down and Detection Kit (ThermoFisher Scientific, MA, USA).

### Immunofluorescence staining

Kidney tissues were preserved in 18% sucrose and subsequently embedded in optimum cutting temperature compound (OCT). A 4-μm section of tissue was mounted on a slide and blocked with 10% normal goat serum for 1 h at room temperature to avoid nonspecific binding. The slides were then incubated with primary antibody (WT-1, Abcam, ab89901, 1:50 dilution) at 4 °C overnight. Afterward, the slides were treated with Alexa Fluor 488-conjugated goat anti-rabbit antibody (Abcam, ab150077, 1:200 dilution) as the secondary antibody. To minimize background fluorescence, anti-fluorescence quenching blockers (Beyotime, Shanghai, P0126) were applied and coverslips were mounted. Finally, the slides were visualized using laser scanning confocal microscopy (LSM) to obtain detailed fluorescence images.

### Periodic Acid-Schiff (PAS) Staining and immunohistochemistry

Kidney tissues were fixed in 4% paraformaldehyde for 24 h, followed by dehydration, and embedded in paraffin. Paraffin sections of 4 μm thickness were cut, and PAS staining was performed according to the instructions provided in the commercial kit (Baso, Zhuhai, BA4080A). For semi-quantitative analysis, 20 glomeruli were randomly selected from each mouse kidney section under a 400 × microscope, and the PAS-positive material (thylakoid matrix) was identified by the purple-red color, while the nucleus was stained blue. Image-J 2.0 image analysis software was utilized to measure the area of the thylakoid matrix and the total glomerular area. The thylakoid stroma index was calculated as thethylakoid stroma area/glomerular area × 100%. Immunohistochemistry for nephrin (Abcam, ab216341, 1:500 dilution) and CXCL16 (Invitrogen, PA5-115,068, 1:100 dilution) on renal sections was performed with streptavidin–biotin peroxidase (SP) kit (ZSGB-BIO, Beijing, China) following standard protocols.

### Transmission electron microscopy.

After removing the mouse kidney, the cortical portion of the kidney was meticulously sliced into thin sections measuring about 2 mm^3^. These sections were promptly submerged in a 2.5% glutaraldehyde electron microscope fixative solution and sent to Electron Microscope Center of Shanghai Medical College, Fudan University.

### Oil red O staining

The oil red O staining procedure for frozen sections of kidneys were immersed in oil red O solution (Oil Red O staining kit; ab150678) for 8 min, followed by incubation in 85% propylene glycol for 1 min to fix the staining. Then, the slides were rinsed with distilled water to remove excess staining and incubated twice in hematoxylin for 2 min to counterstain the nuclei. After gentle rinsing with tap water and distilled water, coverslips were mounted using glycerol to preserve the stained sections.

### Statistics analysis

The experimental results are presented as the means ± Standard error means (SEMs). Statistical analysis was performed with GraphPad Prism software 9 (GraphPad Software Inc., San Diego, CA, USA). Normality of data was assessed using the Shapiro–Wilk test. Two-way analysis of variance (ANOVA) with Tukey's multiple comparisons and Geisser-Greenhouse correction, as well as One-way ANOVA followed by the Tukey’s post hoc analysis, were employed to determine the statistical significance among groups. A significance level of P < 0.05 was used to indicate statistically significant.

## Results

### HQD reduced the ACR and urinary protein levels in db/db mice

**Fig. 1:**
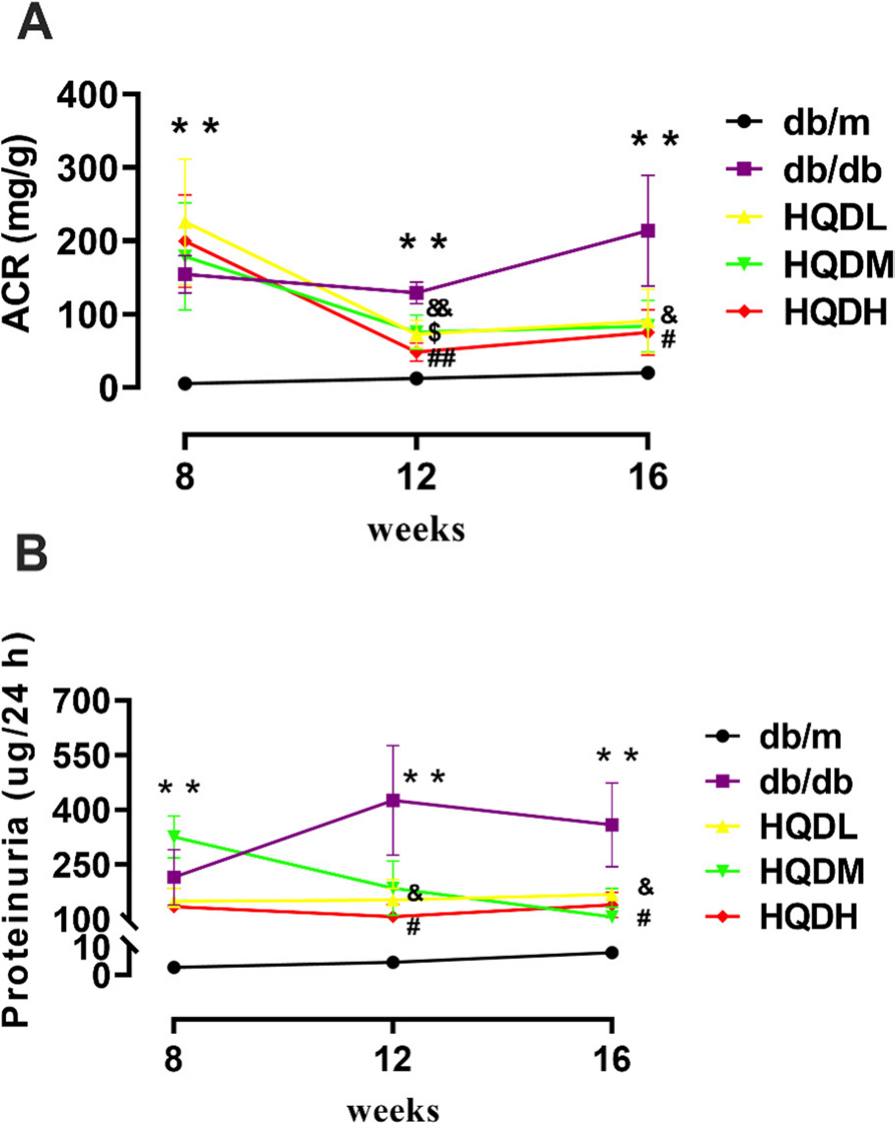
HQD reduced ACR and urinary protein levels in db/db mice. **A**. The level of urine ACR; **B**. 24-h urine protein level. Values are mean ± SEM (*n* = 5–6), **P < 0.01vs. db/m group; &*P* < 0.05 &&*P* < 0.01 HQDL vs. db/db group, $ *P* < 0.05 HQDM vs. db/db group, #*P* < 0.05 ##*P* < 0.01, HQDH vs. db/db group by two-way ANOVA with Tukey's multiple comparisons and Geisser-Greenhouse correction analysis

ACR and urinary albumin levels are highly sensitive indicators for early detection of kidney injury in chronic kidney disease (CKD), and can reflect the severity of kidney damage. Throughout the experiment, ACR and 24-h urinary protein levels in the db/m group remained stable. However, at 8 weeks of age, a significant increase in these two indicators was observed in the db/db group (*P* < 0.01, Fig. 1), indicating successful construction of the DKD model. At 12 weeks of age, although ACR and proteinuria levels were still higher in the HQD group compared to the db/m group, they were significantly lower than those in the db/db group, and this difference became more pronounced at 16 weeks of age (*P* < 0.01, Fig. 1A and B). Random blood glucose levels in the db/db mice continued to increase and were significantly higher than those in the non-diabetic db/m control mice. However, after treatment with different doses of HQD for 8 weeks, there was a trend of downregulation, although it did not reach statistical significance (Figure S1).

### HQD attenuates renal pathological damage and ultrastructural changes in db/db mice

**Fig. 2:**
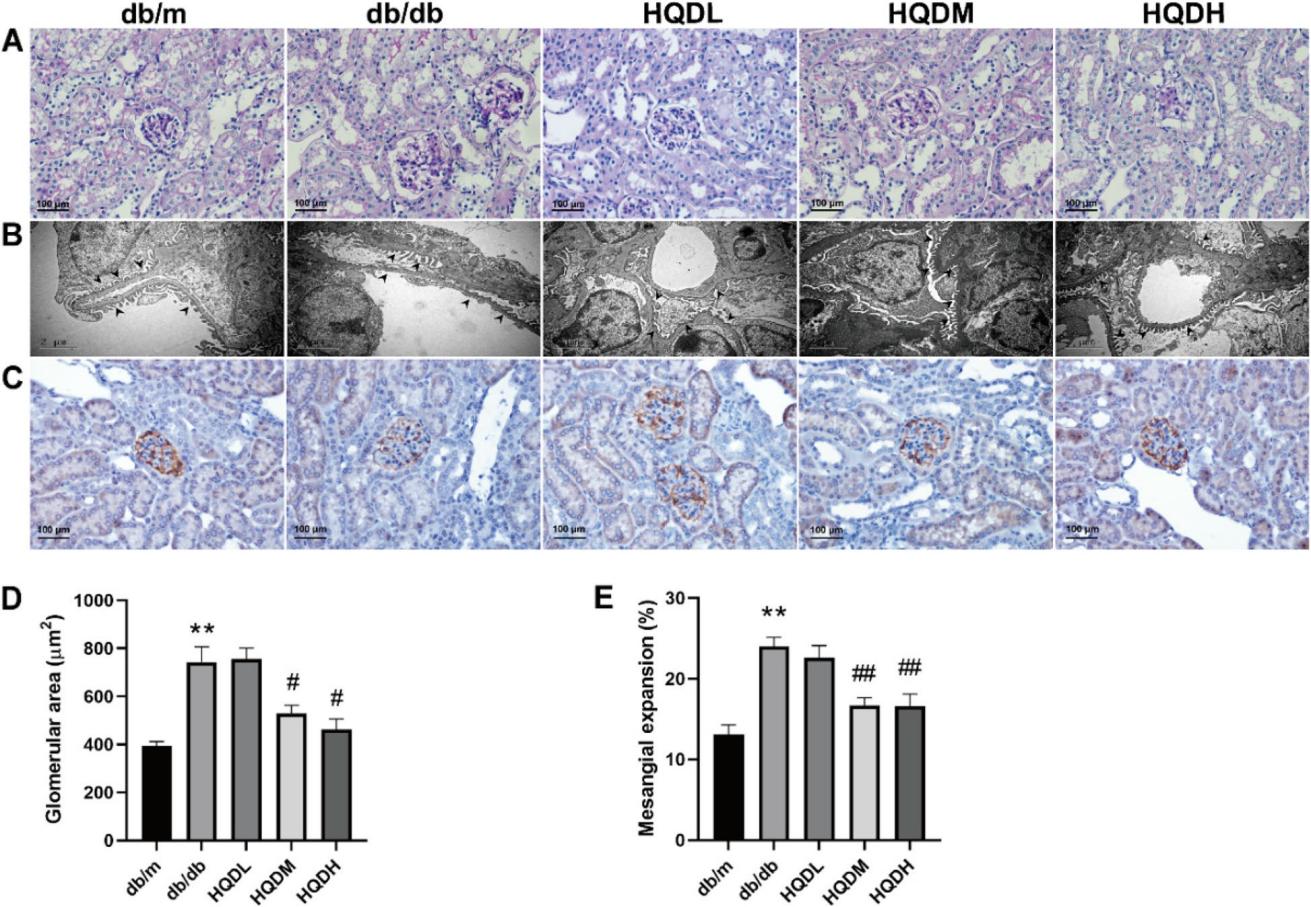
HQD attenuates renal pathological damage and ultrastructural changes in db/db mice. **A**. PAS staining; **B**. SEM images. **C**. Nephrin stained by immunohistochemistry, scale bar, 100 μm; **D**. Glomerular area (μm2). **E**. Mesangial expansion (%). Values are mean ± SEM (*n* = 5–6), **P < 0.01 vs. db/m, #*P* < 0.05, ##*P* < 0.01 vs. db/db by one-way ANOVA with Tukey's post doc analysis

To observe the effect of HQD on renal pathological injuries in db/db mice, PAS staining was performed on paraffin sections of mouse kidney tissue, and the renal pathological conditions of each group were observed by a light microscope. The results showed that compared with db/m mice, db/db mice presented with a significantly increased glomerular volume, widened mesangial areas, and increased proliferation of the mesangial matrix (*P* < 0.01). After treatment with HQD, the above pathological changes were significantly improved (*P* < 0.01, *P* < 0.05) (Fig. 2A, D&E), indicating that HQD could alleviate the pathological damage to kidney tissue in db/db mice. To investigate the effects of HQD on podocyte structure, transmission electron microscopy was performed (Fig. 2B). Podocytes in db/db mice showed broadening, fusion, the disappearance of foot processes, a decrease in the density and number of podocytes, and thickening of the basement membrane. After treatment with HQD, the above phenomena were reduced. A decrease in nephrin (biomarker of podocyte) of glomerulus were observed in model group, which was alleviated in HQD group (Fig. 2C). These results indicate that HQD could maintain the stability of podocyte structure and change the pathological damage to podocytes.

### HQD restored the number of glomerular podocytes in db/db mice

**Fig.3:**
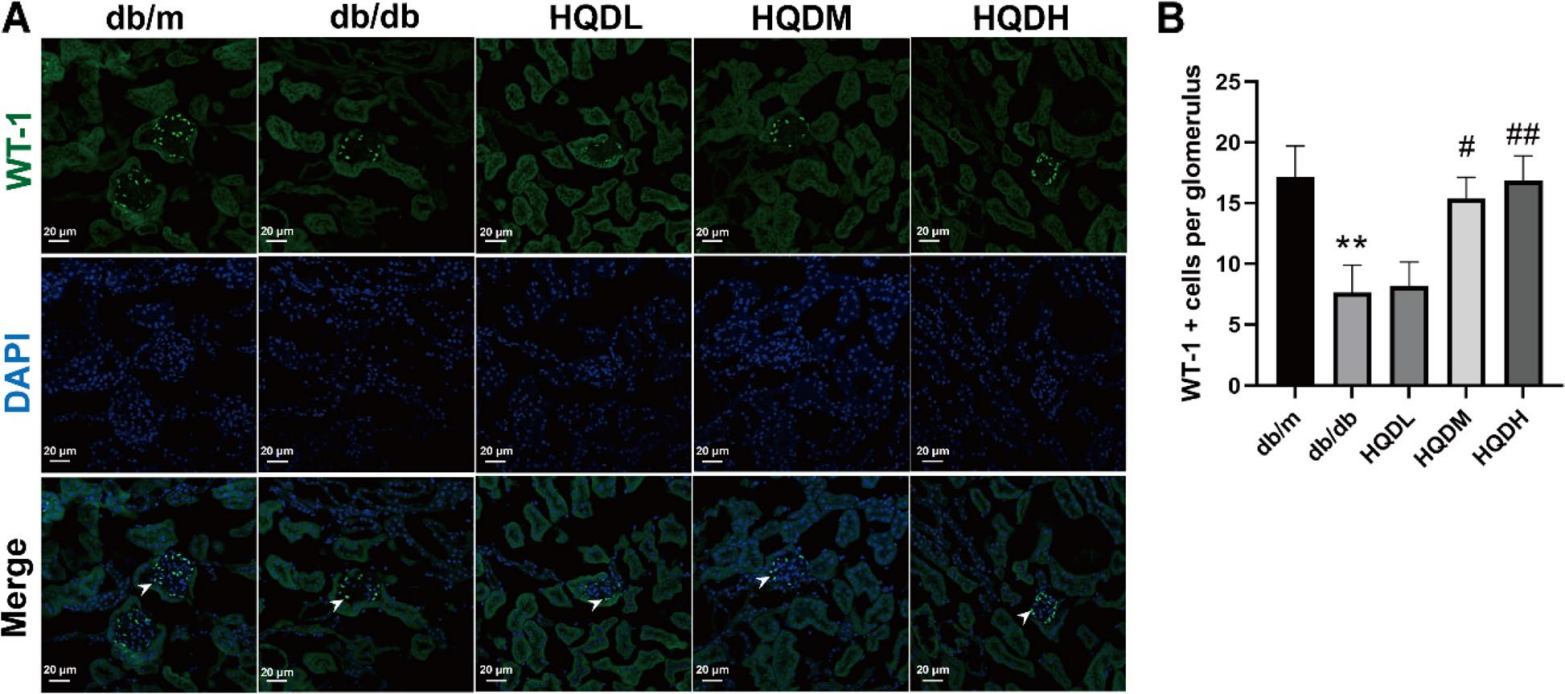
HQD restored the number of glomerular podocytes in db/db mice. **A**. Immunofluorescence shows that the reduced amount of WT-1 in db/db kidneys is significantly restored by HQD. **B**. Statistical results of the number of WT-1 positive cells, Values are mean ± SEM (*n* = 5–6), **P < 0.01 vs. db/m, #*P* < 0.05, ##*P* < 0.01 vs. db/db by one-way ANOVA with Tukey's post doc analysis

In addition to the pathological changes, the loss of glomerular podocytes was also observed, which is known to contribute to the development of DKD. WT1, a podocyte-specific marker, was used to test whether podocyte loss occurs in db/db mice (Fig. 3). As shown in Fig. 3, the number of podocytes was downregulated in db/db mice compared with that in db/m mice, and it was upregulated in HQD-treated db/db mice compared with that in db/db mice (*P* < 0.01, *P* < 0.05).

### Transcriptome study of HQD in db/db mice

**Fig. 4:**
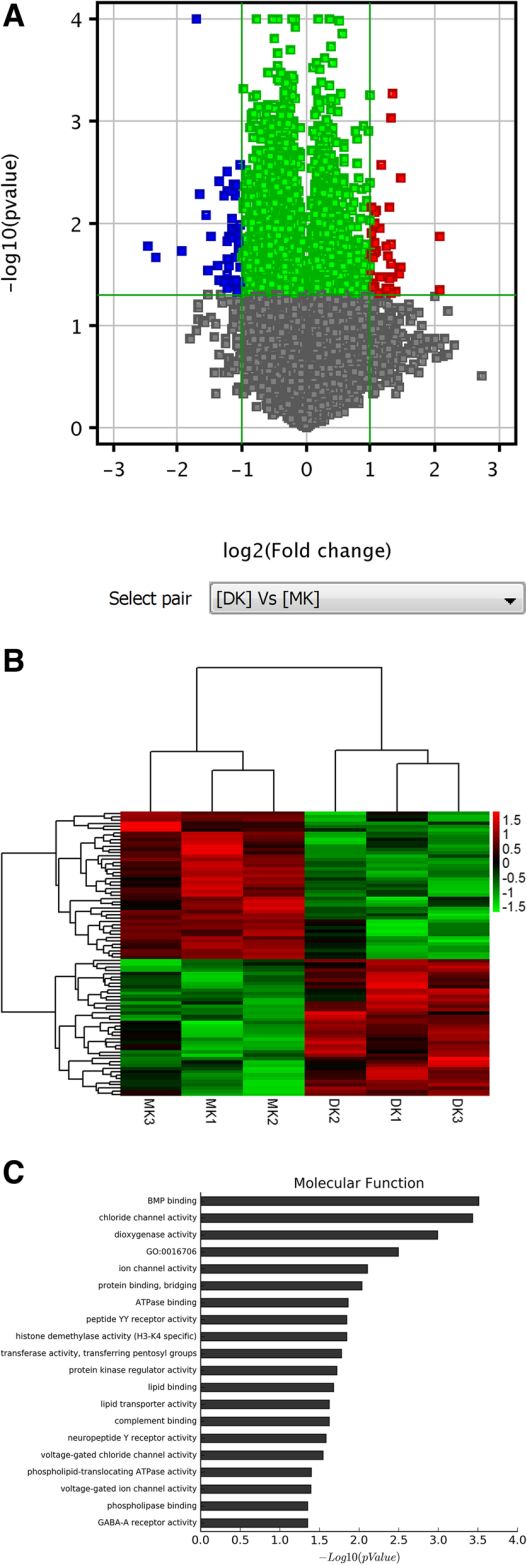
Gene expression profiling Analysis. **A**. Volcano map of differentially expressed mRNAs (*n* = 3). **B**. Cluster heatmap of differentially expressed mRNAs. **C**. The molecular function of differentially expressed mRNAs

To further elucidate the underlying mechanism by which HQD exerts its reno-protective effects, micro-array experiments comparing HQD treated db/db mic or non-treated db/db mice. With fold changes (FC) ≥ 2 or ≤ 0.5 (*P* < 0.05) as the screening criteria, 87 differentially expressed mRNA were obtained, 42 of which in HQD-treated samples were upregulated and 45 were downregulated. Figure 4A is the volcano map of the differentially expressed mRNA. The differentially expressed mRNA was shown in cluster heat map (Fig. 4B). Molecular function of Gene Ontology (GO) enrichment analysis showed that HQD had the greatest effect on the BMP signaling pathway (Fig. 4C).

### HQD restored the expression levels of BMP2, BMP7, and BMPR2

**Fig. 5:**
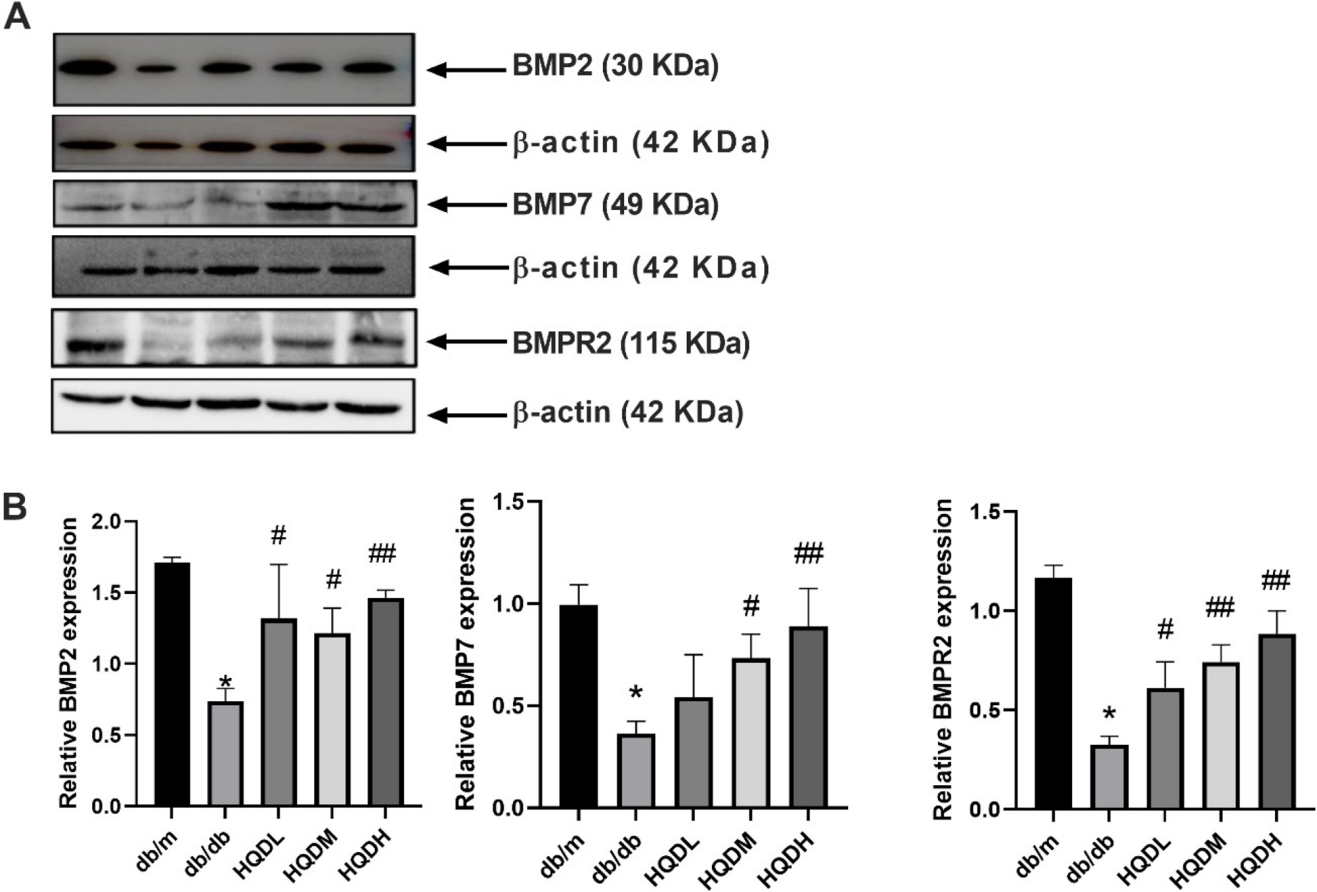
HQD restored the expression levels of BMP2, BMP7, and BMPR2. **A**.**B**. Western blot analysis of renal cortical BMP2, BMP7, and BMPR2 expression levels. Values are mean ± SEM (*n* = 3) *P < 0.05 vs. db/m, #*P* < 0.05, ##*P* < 0.01 vs. db/db by one-way ANOVA with Tukey's post doc analysis

In order to verify the regulatory effect of HQD, the protein expression levels of the BMP family members were detected by Western blot (Fig. 5). Compared with the db/m mice, BMP2 and BMP7 protein expression levels dropped significantly, whereas the expression levels were markedly higher after HQD treatment (*P* < 0.05, *P* < 0.01) (Figs. 5A and B). Further observation showed that HQD could significantly upregulate BMPR2 expression (*P* < 0.05, *P* < 0.01) (Figs. 5A and B). These findings suggest that HQD might play a protective role in the kidneys of db/db mice by regulating BMPR2 and promoting BMP transport.

### HQD downregulated the expression of the Smad protein in the kidneys of db/db mice

**Fig. 6:**
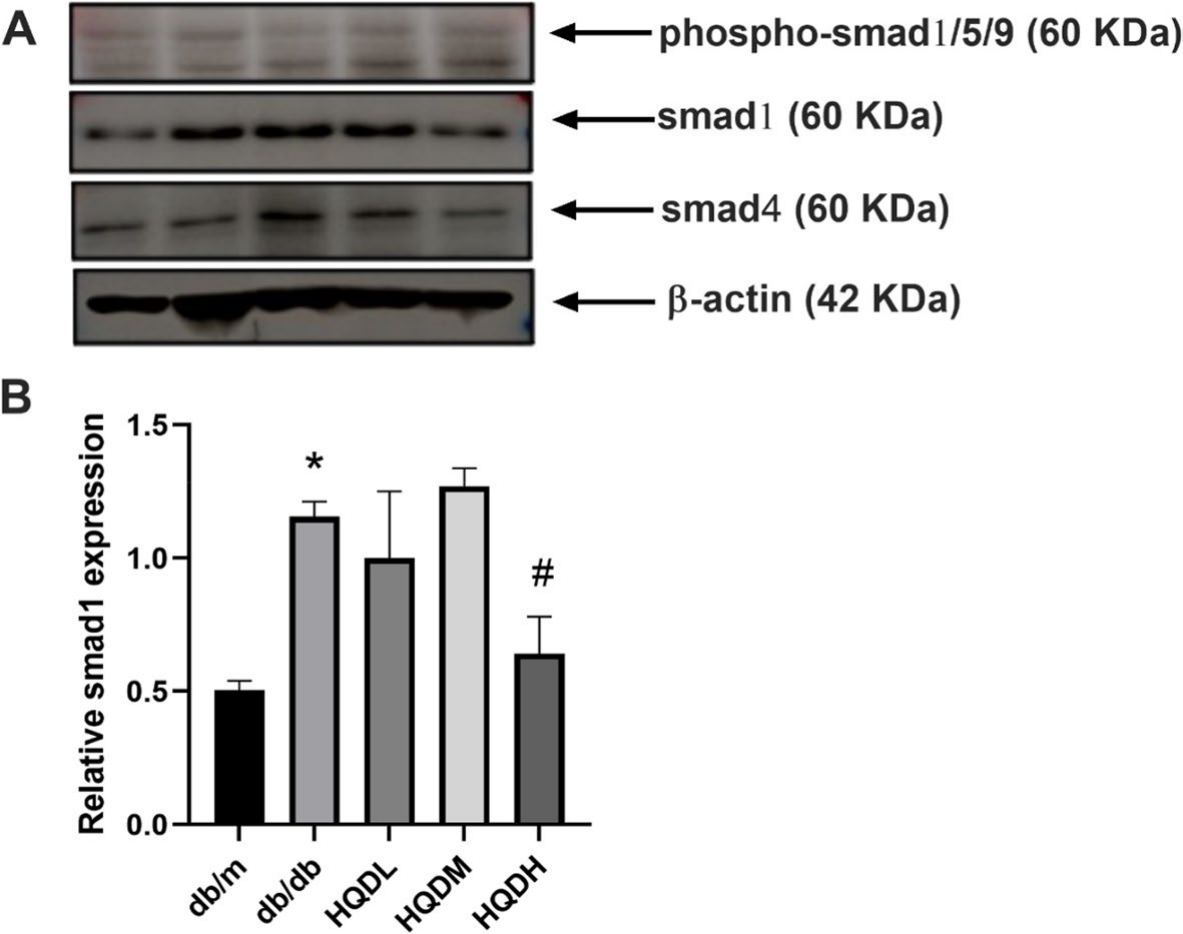
HQD downregulated the expression of the Smad1 protein in the kidneys of db/db mice. **A**. The protein expression levels of P-Smad1/5/9, Smad1 and Smad4 in the kidney cortex by Western blot assay. **B**. Statistical results of the expression level of Smad1, Values are mean ± SEM (*n* = 3), *P < 0.05, compared with db/m group; ##*P* < 0.05, compared with db/db group. *P < 0.05 vs. db/m, #*P* < 0.05 vs. db/db by one-way ANOVA with Tukey's post doc analysis

In order to further explore the mechanism by which HQD may restore the levels of BMPs, the expression of Smad proteins was investigated. Smad1 was significantly increased in the kidneys of db/db mice, and HQD intervention appreciably inhibited its expression (*P* < 0.05, Fig. 6). Notably, there was no significant change in the expression of Smad4, indicating that HQD primarily affects BMP signal transduction through Smad1 rather than Smad4.

### HQD downregulated the expression of ERK and promoted Rap1 binding to GTP

**Fig. 7:**
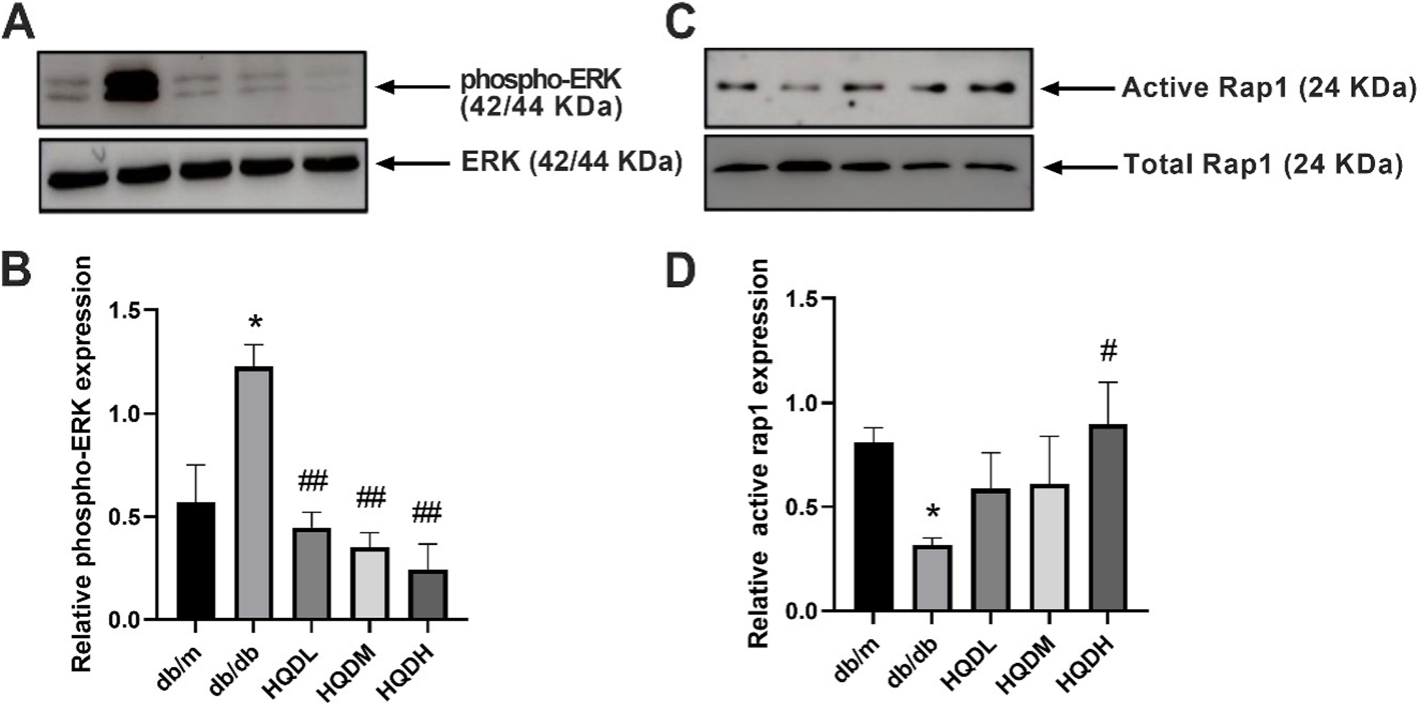
HQD downregulated the expression of ERK and promoted Rap1 binding to GTP. **A**. **B**. The protein expression levels of phospho-ERK in the kidney cortex by Western blot assay. **C**.**D**. In the kidney, the active-Rap1 protein was enriched using the active-rap1 pull-down kit, and alterations were detected by Western blot. Values are mean ± SEM (*n* = 3), *P < 0.05 vs. db/m, #*P* < 0.05 vs. db/db by one-way ANOVA with Tukey's post doc analysis

Subsequently, the phosphorylation status of ERK and the activity of Rap1 were assessed. The protein level of phospho-ERK in the kidneys of db/db mice was markedly increased, and its expression was effectively restored by HQD (*P* < 0.05, *P* < 0.01) (Fig. 7A&B). Rap1 acts as a molecular switch by binding GDP and GTP to switch between its inactive and activated states. The activity of Rap1 was significantly decreased in db/db mice, while HQD treatment restored the expression of Rap1-GTP in the kidneys of db/db mice to normal levels (*P* < 0.05, Fig. 7C&D).

### HQD ameliorates lipid metabolism disorders in db/db mice

**Fig. 8:**
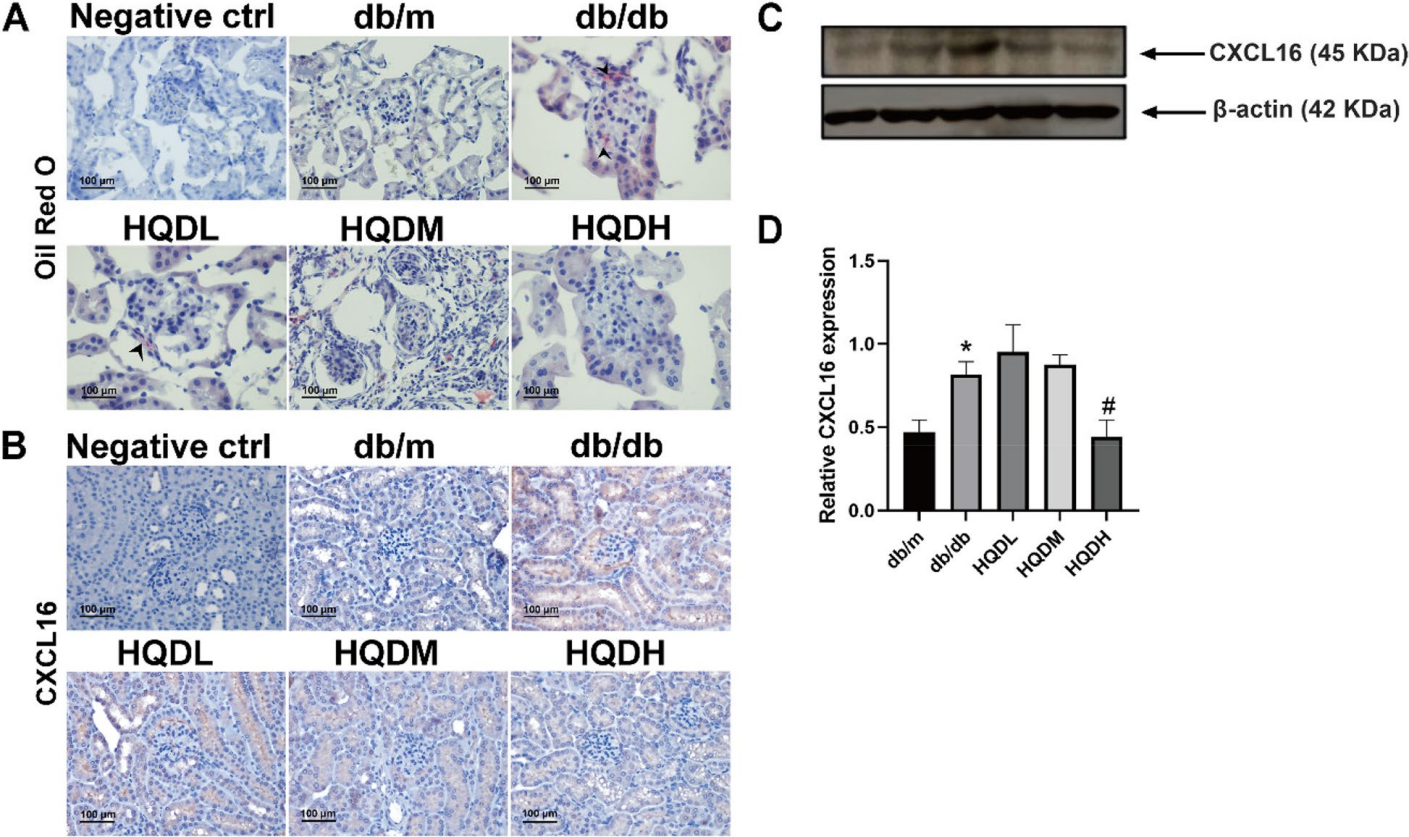
HQD ameliorates lipid metabolism disorders in db/db mice. **A**. Frozen sections of mouse kidneys were stained with Oil Red O (*n* = 5). **B**. IHC staining of mouse paraffin sections for CXCL16 (*n* = 5). **C**.**D** The protein expression levels of CXCL16 in the kidney cortex by Western blot assay (*n* = 3). *P < 0.05 vs. db/m, #*P* < 0.05 vs. db/db by one-way ANOVA with Tukey's post doc analysis

Diabetes mellitus is a disease characterized by metabolic abnormalities. Prolonged hyperglycemia associated with diabetes eventually leads to lipid metabolism disorders. In this study, it was observed that HQD significantly improved renal lipid deposition in db/db mice, as evidenced by Oil Red O staining (Fig. 8A). CXCL16, a key intermediate mediator of lipid accumulation, localizes in both tubules and glomeruli. In diabetic mice, the intensity of CXCL16 staining in glomerular was significantly increased. However, HQD dose-dependently decreased the expression of CXCL16 in both glomeruli and tubules (Fig. 8B). Western blots analysis further confirmed these findings (*P* < 0.05, Fig. 8C&D).

## Discussion

DKD is one of the most devastating and prevalent chronic microvascular complications of diabetes and a major contributor to ESRD and death. Experimental studies have demonstrated that TCM can effectively improve DKD [30, 31], but the relevant pharmacological mechanisms underlying these effects are not sufficiently understood. The formulation of HQD has been supported by years of clinical and basic research, confirming its suitability and reliability in protecting against CKD [20, 21, 23, 24]. In this study, the therapeutic efficacy of HQD was associated with improvements of BMP/Smad signaling pathway, which is consistent with micro-assay results. Furthermore, HQD was able to regulate abnormal lipid levels that occur during long-standing chronic hyperglycemia, thus restoring lipid metabolism balance. These results suggest that HQD may be superior to many current drugs due to its multiple activities, which can potentially alleviate the clinical symptoms of DKD.

HQD treatment induces a distinct gene expression pattern that is mainly related to BMP signaling. BMPs belong to the TGF-β superfamily, which is a class of highly conserved functional proteins. In BMP4 -/- transgenic mice, which lack BMP4 secretion by podocytes, pathological changes such as glomerular microaneurysms, collapsed glomerular capillaries, enlarged glomerular capsules, and reduced normal proximal tubules have been observed, indicating that BMP of podocyte origin has an important function in glomerular capillary formation [32]. BMP4 has also been shown to regulate the deposition of the extracellular matrix in the glomerular thylakoid region of DKD mice, which exacerbates glomerular lesions [33]. In the early formation of type I diabetes in rats, the expression of renal BMP7 and its receptors was reduced or lost. However, pharmacological intervention has been shown to improve renal function, reduce fibronectin expression, and reverse the expression of both BMP7 and its receptor compared to DKD rats [34]. Furthermore, BMP7 has been found to protect thylakoid cells from high glucose-induced oxidative stress, thereby delaying DKD [35]. BMPRs are a group of serine/threonine kinase receptors with abundant cysteine extracellular structures, single-chain transmembrane regions and intracellular kinase regions. These receptors include type I and type II receptors, where type I receptors are the downstream activating components of type II receptors [36]. Gene microarray data has showed that HQD treatment in db/db mice robustly alters glomerular BMP binding pathway-related signaling. Further analysis of renal BMP-related proteins in db/db mice and discovered that HQD could effectively restore the expression levels of BMP2 and BMP7, and dramatically elevate the expression of BMPR-2. This finding suggests that HQD may protect against DKD by regulating BMPR-2 and promoting BMP2 and BMP7 transport.

BMPs are part of the kinase transduction system, where ligand-receptor binding leads to phosphorylation of the threonine-serine-rich (GS) structural domain in the cell membrane of the type I receptor by the type II receptor. This phosphorylation event activates the Smad protein, which further transduces the biological message of BMP [36]. The classic BMP pathway transduction is dependent on the modulation of Smad proteins, which are regulated by various molecular mechanisms, affecting signaling at different levels including ligands, receptors, and signal transduction factors. Deletion of BMPR-2 inhibits the downstream Smad1/5 phosphorylation, which in turn inhibits BMP signaling [34]. Previous studies found that HQD could participate in regulating renal interstitial fibrosis through the Smad signaling pathway [21]. In the current study, it was observed that renal Smad protein expression levels were elevated in db/db mice. However, the increased expression of Smad1 protein in db/db mice was effectively suppressed by treatment with HQD. Meanwhile, HQD did not affect Smad4 expression. This discrepancy may be attributed to the fact that Smad4 is a coregulated component of the TGF-β and BMP signaling pathways, and thus may exert opposing effects on the kidney. It has been suggested that Smad4 deficiency inhibits renal fibrosis, but it can also result in increased kidney inflammation [37].

The Ras-like small GTPase Rap1 functions as a molecular switch by binding GTP or GDP to switch between its activation and inactivation states, and the absence of Rap1 signaling is directly related to the integrity of the podocyte [38]. Additionally, ERK, a member of the MAPK family, has been shown to regulate the phosphorylation of Smad1 in pulmonary artery smooth muscle cells, leading to the promotion of BMP entry into the nucleus [39]. Interestingly, decreased phosphorylation of ERK has been observed in the glomeruli of FSGS patients, which correlates with the inactivation of Rap1GTP [14]. However, treatment with HQD has been shown to restore the phosphorylation level of ERK and the binding of Rap1 to GTP. It’s worth noting that the results may be influenced by other cells in the kidney, such as endothelial cells and mesangial cells and other resident cells, and further in vitro studies are needed to explore the relationship between ERK and Rap1 activation specifically in podocytes.

In patients with DKD, disruptions in lipid metabolism are commonly observed. BMPs are also known to be involved in maintaining metabolic processes [19], as evidenced by increased expression of BMP2 in human adipose tissue and studies in mice showing that BMP2 is associated with the production of white adipose tissue [40, 41]. Hata et al. demonstrated that BMP2 induces adipogenesis in C3H10T1/2 cells through the activation of peroxisome proliferator-activated receptor γ (PPARγ) by the Smad and p38 MAPK signaling pathways [42]. On the other hand, BMP7 is closely associated with brown adipogenesis, and deletion of BMP7 in mice results in a significant reduction in brown adiposity at birth [43]. In a study involving obese db/db mice, intraperitoneal injection of BMP7 resulted in a distinct reduction in body weight and body fat compared to controls one month later [44]. In this study, db/db mice showed significant glomerular lipid accumulation compared to controls. HQD improved the extent of lipid accumulation in db/db mice while modulating BMP. As HQD consists of seven herbs and has multiple pathways according to its complicated components and the results of the micro-assay study. Transcriptomic analysis revealed that HQD had the greatest effect on the BMP signaling pathway. ERK, Smad1, BMPs were regulated by HQD in the kidney in db/db mice. However, further studies, such as using transgenic mice or ERK inhibitors, are needed to elucidate the key mechanism underlying the effects of HQD.

## Conclusions

In general, HQD demonstrated a significant protective effect against DKD. This beneficial effect may be attributed to several mechanisms. Firstly, HQD was found to promote the transcription of BMPs and their downstream target genes by upregulating BMPR-II expression. Moreover, HQD was shown to regulate the phosphorylation of ERK and the expression of Smad1 by promoting the binding of Rap1 to GTP, thus modulating the fibrotic signaling pathways. Furthermore, HQD was also found to have a notable role in regulating lipid metabolism dysfunction in DKD, providing a new avenue for future research on HQD. The exact mechanisms underlying this effect are not yet fully understood, but HQD has been shown to modulate lipid metabolism-related gene expression and improve lipid profiles in animal studies, suggesting a potential role in ameliorating lipid abnormalities commonly associated with DKD.

## Supplementary Information

**Additional file 1.** 

**Additional file 2.**

## Data Availability

The datasets generated during the current study are available in NCBI repository using the accession number GSE225477.

## Abbreviations

ACEIs: Angiotensin-converting enzyme inhibitors
ACR: Albumin/creatine ratio
ARBs: Angiotensin receptor blockers
BMPs: Bone morphogenetic proteins
CKD: Chronic kidney disease
CMC: Carboxymethylcellulose
CV: Coefficients of variance
DKD: Diabetic kidney disease
ECL: Enhanced chemiluminescence
ERK: Extracellular regulated protein kinase
ESRD: End-stage renal disease
FC: Fold changes
GO: Gene Ontology
HQD: Huangqi decoction
LSM: Laser scanning confocal microscopy
MAPK: Mitogen-activated protein kinase
OCT: Optimum cutting temperature compound
PAS: Periodic Acid-Schiff
RIN: RNA integrity number
SEMs: Standard error means
TCM: Traditional Chinese medicine
TGF-β: Transforming growth factor
