# Supplementary material for: Huangqi decoction ameliorates kidney injury in db/db mice by regulating the BMP/Smad signaling pathway
Source: BMC Complement Med Ther. 2023 Jun 26;23:209. doi: 10.1186/s12906-023-04029-1 (PMC10294356; doi:10.1186/s12906-023-04029-1)

**Supplementary Figures S1**

**Figure. S1 Effect of HQD on blood glucose levels in db/db mice. (**A). Changes in random blood glucose levels in the five groups of mice over the whole experiment. Values are mean ± SEM（n=5-6）, **P<0.01, compared with the db/m group by two-way ANOVA with Tukey's multiple comparisons.


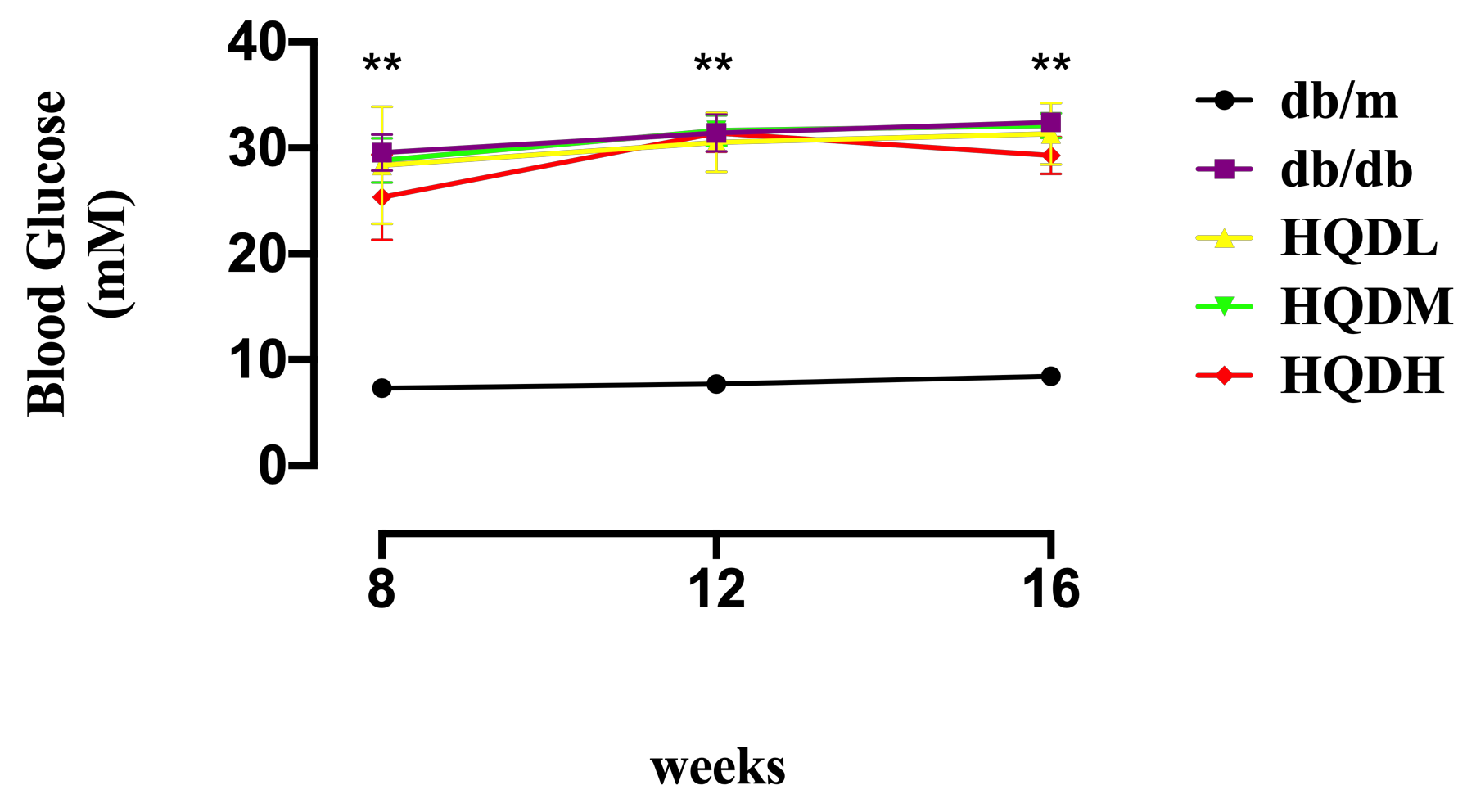

Supplement: Supplementary file 1 — Additional file 1. [file 12906_2023_4029_MOESM1_ESM.docx]
