# Supplementary material for: Huangqi decoction ameliorates kidney injury in db/db mice by regulating the BMP/Smad signaling pathway
Source: BMC Complement Med Ther. 2023 Jun 26;23:209. doi: 10.1186/s12906-023-04029-1 (PMC10294356; doi:10.1186/s12906-023-04029-1)
Supplement: Supplementary file 2 — Additional file 2. [file 12906_2023_4029_MOESM2_ESM.doc]

**LC-MS/MS analysis**

An LC-MS/MS method was used to determine the 9 major components (Schisandrin, Liquiritin, Acteoside, Glycyrrhizic acid, Astilbin, Astragaloside IV, Ruscogenin, Catalpol, and 3, 29-Dibenzoyl rarounitriol) in HuangQi decoction. Chromatography was performed on an Agilent 1200 LC system (Agilent Technologies Inc., USA). Schisandrin, Liquiritin, Acteoside, Glycyrrhizic acid, Astilbin, Astragaloside IV) were separated on a Thermo Betasil C_18_ column (50 × 2.1 mm, i.d., 5.0 µm). The mobile phase consisted of 0.1% formic acid in water (A) and 0.1% formic acid in acetonitrile (B) with gradient elution at the flow rate of 0.6 ml/min. The gradient elution program was set as follow: 0-0.1 min 5% B, 0.1-1.3 min 5-95% B, 1.3-1.7 min 95% B, 1.7-1.8 min 95-5% B, 1.8-3.0 min 5% B. Ruscogenin was separated on a Thermo Betasil C_18_ column (50 × 2.1 mm, i.d., 5.0 µm). The mobile phase consisted of 0.1% formic acid in water (A) and 0.1% formic acid in acetonitrile (B) with gradient elution at the flow rate of 0.6 ml/min. The gradient elution program was set as follow: 0-0.1 min 2% B, 0.1-1.3 min 2-98% B, 1.3-1.7 min 98% B, 1.7-1.8 min 98-2% B, 1.8-3.0 min 2% B. Catalpol was separated on a Phenomenex, Gemini, C18, 50 × 4.6 mm mm, 5µm). The mobile phase consisted of 0.1% formic acid in water (A) and 0.1% formic acid in acetonitrile (B) with gradient elution at the flow rate of 0.8 ml/min. The gradient elution program was set as follow: 0-0.1 min 30% B, 0.1-1.3 min 30-95% B, 1.3-1.7 min 95% B, 1.7-1.8 min 95-30 B, 1.8-3.0 min 30% B. 3, 29-Dibenzoyl rarounitriol was separated on a Thermo Hypersil C_18_ column, 50 × 2.1 mm mm, 5µm). The mobile phase consisted of 10 mM ammonium acetate and 0.1% formic acid in water (A) and methanol (B) with gradient elution at the flow rate of 0.6 ml/min. The gradient elution program was set as follow: 0-0.1 min 5% B, 0.1-1.3 min 5-98% B, 1.3-1.7 min 98% B, 1.7-1.8 min 98-5 B, 1.8-3.0 min 5% B.

MS detection was conducted on QTRAP 5500 mass spectrometer (Applied Biosystem Sciex, Ont., Canada) equipped with a TurboSpray ionization source. Optimized parameters were as follws: curgain gas, gas 1 and gas 2 were 15, 40 and 30 psi respectively; source temperature 550 °C; spray voltage 5500 V. The detections were performed in multiple reaction monitoring (MRM) mode, and ion transitions were set at *m/z* 433.4→*m/z* 384.3, *m/z* 417.4→*m/z* 255.1, *m/z* 449.3→*m/z* 150.7, *m/z* 684.5→*m/z* 527.5, *m/z* 623.2→*m/z* 161, *m/z* 821.5→*m/z* 351.1, *m/z* 829.4→*m/z* 783.5, *m/z* 431.5→*m/z* 287.3, and *m/z* 361.4→*m/z* 169 for Schisandrin, Liquiritin, Astilbin, 3, 29-Dibenzoyl rarounitriol, Acteoside, Glycyrrhizic acid, Astragaloside IV, Ruscogenin, and Catalpol, respectively.

**Result**

In this study nine major components were quantified using LC-MS/MS method. Except 3, 29-Dibenzoyl rarounitriol, all other eight components were detected in HuangQi decoction and further quantified. The extracted ion chromatograms of the detected components in HuangQi decoction were shown in Fig. S1. Per gram of dry extract contained 254.2 µg of Schisandrin, 1074.2 µg of Liquiritin, 99.6 µg of Acteoside, 1215.8 µg of Glycyrrhizic acid, 4.4 µg of Ruscogenin, 0.2 µg of Astilbin, 440.2 µg of Astragaloside IV and 4.3 µg of Catalpol.

B

C

D

E

F

G

H

A

I

**Fig. S2.** The extracted ion chromatograms of the detected components in HD (A: Schisandrin, B: Liquiritin, C: Acteoside, D: Glycyrrhizic acid, E: Astilbin, F: Astragaloside IV, G: Ruscogenin, H: Catalpol, and I: 3,29-Dibenzoyl rarounitriol)
